# Effects of Antioxidant in Adjunct with Periodontal Therapy in Patients with Type 2 Diabetes: A Systematic Review and Meta-Analysis

**DOI:** 10.3390/antiox10081304

**Published:** 2021-08-18

**Authors:** Koji Mizutani, Prima Buranasin, Risako Mikami, Kohei Takeda, Daisuke Kido, Kazuki Watanabe, Shu Takemura, Keita Nakagawa, Hiromi Kominato, Natsumi Saito, Atsuhiko Hattori, Takanori Iwata

**Affiliations:** 1 Department of Periodontology, Graduate School of Medical and Dental Sciences, Tokyo Medical and Dental University (TMDU), Bunkyo, Tokyo 113-8549, Japan; mizuperi@tmd.ac.jp (K.M.); tak2peri@tmd.ac.jp (K.T.); takemura.peri@tmd.ac.jp (S.T.); nakagawa.peri@tmd.ac.jp (K.N.); komiperi@tmd.ac.jp (H.K.); natuperi@tmd.ac.jp (N.S.); iwata.peri@tmd.ac.jp (T.I.); 2 Department of Conservative Dentistry and Prosthodontics, Faculty of Dentistry, Srinakharinwirot University, Bangkok 10110, Thailand; prima@g.swu.ac.th; 3 Department of Oral Diagnosis and General Dentistry, Dental Hospital, Tokyo Medical and Dental University (TMDU), Tokyo 113-8549, Japan; kidperi@tmd.ac.jp; 4 Department of Biology, College of Liberal Arts and Sciences, Tokyo Medical and Dental University (TMDU), Chiba 272-0827, Japan; kwatperi@tmd.ac.jp (K.W.); ahattori.las@tmd.ac.jp (A.H.)

**Keywords:** antioxidant, diabetes, melatonin, meta-analysis, periodontal disease, systematic review

## Abstract

This review investigated whether the adjunctive use of antioxidants with periodontal therapy improves periodontal parameters in patients with type 2 diabetes. A systematic and extensive literature search for randomized controlled trials (RCTs) conducted before April 2021 was performed on the PubMed, Cochrane Library, and Web of Science databases. The risk of bias was assessed using the Cochrane risk-of-bias tool. A meta-analysis was performed to quantitatively evaluate the clinical outcomes following periodontal therapy. After independent screening of 137 initial records, nine records from eight RCTs were included. The risk-of-bias assessment revealed that all RCTs had methodological weaknesses regarding selective bias, although other risk factors for bias were not evident. This meta-analysis of two RCTs showed that periodontal pocket depths were significantly reduced in the groups treated with combined non-surgical periodontal therapy and melatonin than in those treated with non-surgical periodontal therapy alone. The present systematic review and meta-analysis suggest that the adjunctive use of melatonin, resveratrol, omega-3 fatty acids with cranberry juice, propolis, and aloe vera gel with periodontal therapy significantly improves periodontal disease parameters in patients with type 2 diabetes, and melatonin application combined with non-surgical periodontal therapy might significantly reduce periodontal pocket depth. However, there are still limited studies of melatonin in combination with non-surgical periodontal therapy in Type 2 diabetic patients, and more well-designed RCTs are required to be further investigated.

## 1. Introduction

Periodontitis is an inflammatory and infectious disease caused by dental plaque [1,2]. Considerable evidence has indicated a relationship between periodontitis and non-communicable diseases such as diabetes, heart diseases [3,4], and chronic kidney disease [5,6,7]. In particular, type 2 diabetes and periodontal disease are known to be bidirectionally related. The prevalence of periodontal disease is high in diabetic patients [8,9], and it is generally agreed that periodontal treatment significantly downregulates glycated hemoglobin A1c (HbA1c) levels [10]. Periodontal treatment improves glycemic control by eliminating the bacterial infection that causes periodontal disease, thereby suppressing the local inflammatory response in the periodontal tissue and improving insulin resistance. More recently, it has been reported that glycemic control independently affects periodontal inflammation, regardless of oral hygiene conditions [11]. Therefore, further research on internal host reaction-related pathways, rather than on inflammation control against bacterial infections [12], is needed for both diabetes and periodontal disease.

Diabetes increases oxidative stress, which in turn can worsen both insulin action and secretion, thereby accelerating the progression to overt the disease [13,14]. Studies suggested increased oxidative stress markers *Nox1*, *Nox2*, *Nox4*, and *p47* in diabetes model rats [14,15] and plasma d-8-iso prostaglandin F2a (d-8-iso) in diabetic patients [16]. Elevation of reactive oxygen species (ROS) might play one of the most critical roles in establishing, progressing periodontitis [17,18]. An increase in ROS generates an impaired bone formation by activating forkhead box transcription factors (FoxO) and bone resorption by decreasing Wnt signaling [19]. Furthermore, malondialdehyde (MDA), nitric oxide, total oxidant status (TOS), and 8-hydroxy-deoxyguanosine levels were increased in the saliva and gingival crevicular fluid of patients with periodontitis [20]. In vitro study, the antioxidant *N*-acetyl-l-cysteine (NAC) could restore cell migration, proliferation, and cell cytotoxicity in human gingival fibroblasts grown at high glucose concentrations. Thus, it can be hypothesized that reducing oxidative stress through antioxidant application would be effective in treating diabetes and periodontal disease [21]. In clinical practice, some studies have shown that topical or systemic use of antioxidants in periodontal therapy can reduce local inflammation [22]. For example, the adjunctive use of vitamin C in periodontal therapy has been previously reported [23], and administration of lycopene [24] and vitamin E [25] have been analyzed for periodontal treatment in patients without medical disorders. The generally recognized treatments to reduce PPD include non-surgical therapy such as SRP [26] or periodontal surgery [27]. In fact, SRP improved PPD in the control groups of the studies included in this review. Previous systematic reviews have shown that combinations of different treatments with SRP do not provide additional benefits to the primary outcome of periodontal treatment [27,28]. However, combination therapy may be effective in patients with type 2 diabetes, which is associated with the progression of periodontal disease [29] and impaired postoperative healing [9]. Thus, the combination of SRP and antioxidant supplements may have clinical benefits. However, few meta-analyses of the combined use of antioxidants and periodontal therapy in patients with diabetes exist in the literature.

Melatonin is an endogenous hormone that is mainly released from the pineal gland in response to darkness [30]. It is involved in several biological functions, including immune responses [31], anti-inflammatory processes [32], bone homeostasis [33], and energy metabolism [34]. Moreover, its functions as an endogenous free-radical scavenger and broad-spectrum antioxidant are well established [32,35]. Mammals have two melatonin receptor subtypes, namely *MTNR1A* (MT1) and *MTNR1B* (MT2). Interestingly, the MT2 variant is significantly associated with increased fasting blood glucose levels, reduced early insulin response to glucose, and increased risk of type 2 diabetes mellitus [36,37]. In contrast, the administration of melatonin for 3 months improved glycemic control in patients with type 2 diabetes [38]. Furthermore, a combination of melatonin and zinc acetate with metformin resulted in improvements in the lipid profile and vascular complications in patients with poorly controlled type 2 diabetes compared to administration of metformin alone [39]. Thus, melatonin may have therapeutic potential in the treatment of diabetes mellitus and its complications. Previously, topical melatonin application adjunctive to periodontal treatment significantly decreased GI and PPD, and significantly decreased IL-6 and serum C-reactive protein levels in patients with diabetes and periodontal disease [40].

This study aimed to conduct a systematic review to address the following clinical question: Does the adjunctive use of antioxidants such as melatonin, resveratrol and ascorbic acid with periodontal therapy improve the status of periodontal parameters in patients with type 2 diabetes? In addition, a meta-analysis of periodontal treatment outcomes was performed to determine whether specific antioxidants were applied using the same evaluation methods in multiple studies.

## 2. Materials and Methods

A search strategy was applied according to the preferred reporting items for systematic reviews and meta-analysis (PRISMA) protocol [41,42].

### 2.1. Search Strategy

An extensive literature search was performed using the PubMed, Cochrane Library, and Web of Science databases to summarize the currently available knowledge and answer the aforementioned clinical question by isolating randomized controlled trials (RCTs) investigating the effects of antioxidants with periodontal treatment in patients with diabetes prior to 6 April 2021. The search terms related to antioxidants were set according to the previous studies [43,44,45,46,47,48]. The search terms used in PubMed are listed below: 

(periodontal diseases [MeSH Terms] OR periodontal disease[Title/Abstract] OR periodontium [MeSH Terms] OR periodontics [MeSH Terms] OR periodontitis [Title/Abstract] OR periodontitis [MeSH Terms]) AND (“diabetes mellitus”[MeSH Terms] OR “diabetes insipidus”[MeSH Terms] OR “diabet*”[Title/Abstract] OR “dm 1”[Title/Abstract] OR “dm i”[Title/Abstract] OR “dm 2”[Title/Abstract] OR “dm ii”[Title/Abstract] OR “glycated hemoglobin a”[MeSH Terms] OR “a1c”[Title/Abstract] OR “hb a1c”[Title/Abstract] OR “hba1c”[Title/Abstract] OR “blood glucose”[MeSH Terms] OR “blood sugar”[Title/Abstract] OR ((“glucose”[Title] OR “sugar”[Title]) AND (“level”[Title] OR “control”[Title])) OR “hyperglycemia”[MeSH Terms] OR “hypoglycemia”[MeSH Terms] OR “glycemi*”[Title/Abstract] OR “glycaemi*”[Title/Abstract] OR “hyperglyc*”[Title/Abstract] OR “hypoglyc*”[Title/Abstract]) AND (“Antioxidants”[MeSH Terms] OR “Antioxidants”[Title/Abstract] OR “antioxidant effect”[Title/Abstract] OR “effect antioxidant”[Title/Abstract] OR “antioxidant effect”[Title/Abstract] OR “antioxidant effect”[Title/Abstract] OR “effect antioxidant”[Title/Abstract] OR “antioxidant effects”[Title/Abstract] OR “antioxidant effects”[Title/Abstract] OR “effects antioxidant”[Title/Abstract] OR “antioxidant effects”[Title/Abstract] OR “effects antioxidant”[Title/Abstract] OR “Resveratrol”[Title/Abstract] OR “Tea”[MeSH Terms] OR “Tea”[Title/Abstract] OR “ascorbic acid”[MeSH Terms] OR “ascorbic acid”[Title/Abstract] OR “acid ascorbic”[Title/Abstract] OR “l ascorbic acid”[Title/Abstract] OR “acid l ascorbic”[Title/Abstract] OR “l ascorbic acid”[Title/Abstract] OR “vitamin c”[Title/Abstract] OR “vitamin e”[MeSH Terms] OR (“aloe”[MeSH Terms] OR “aloe”[Title/Abstract] OR (“aloe”[Title/Abstract] AND “vera”[Title/Abstract]) OR “aloe vera”[Title/Abstract]) OR (“melatonin”[MeSH Terms] OR “melatonin”[Title/Abstract] OR “melatonins”[Title/Abstract] OR “melatonine”[Title/Abstract] OR “melatonins”[Title/Abstract]) OR (“propolis”[MeSH Terms] OR “propolis”[Title/Abstract]) OR (“coenzyme q10”[Supplementary Concept] OR “coenzyme q10”[All Fields] OR “coenzyme q10”[All Fields] OR “ubiquinone”[MeSH Terms] OR “ubiquinone”[All Fields] OR (“coenzyme”[All Fields] AND “q10”[All Fields])) OR (“polyphenol s”[All Fields] OR “polyphenoles”[All Fields] OR “polyphenolic”[All Fields] OR “polyphenolics”[All Fields] OR “polyphenols”[MeSH Terms] OR “polyphenols”[All Fields] OR “polyphenol”[All Fields]) OR (“curcumin”[MeSH Terms] OR “curcumin”[All Fields] OR “curcumin s”[All Fields] OR “curcumine”[All Fields] OR “curcumins”[All Fields])). 

A similar search strategy was applied to all the databases. Additional electronic searches were performed in the Journal of Periodontology, Journal of Clinical Periodontology, Journal of Periodontal Research, and Antioxidants, to increase the likelihood of identifying relevant papers [49].

### 2.2. Study Selection

In the first stage, the titles and abstracts of all retrieved articles were screened for potentially eligible studies. Full-length articles of the identified studies were examined in detail according to the eligibility criteria for inclusion in this review. Two reviewers (RM and KM) independently performed the screening process. In case of a disagreement between the reviewers, a consensus was reached through discussion. The following studies were included:

1. RCTs examined the efficacy of antioxidants on periodontal parameters in patients with both type 2 diabetes and periodontitis.

2. Studies with periodontal treatment with non-surgical therapy, such as scaling and root planing (SRP), or surgical therapies such as flap procedures, as interventions.

3. Studies in which participants were allocated to experimental and placebo/control groups.

4. Studies with outcome variables including clinical parameters for periodontitis such as probing pocket depth (PPD), clinical attachment level (CAL), and bleeding on probing (BOP).

5. Studies published in English.

The exclusion criteria were as follows:

1. Review articles, case reports, descriptive studies, opinion articles, abstracts, animal experiments, and in vitro studies.

2. Clinical studies conducted on participants with diabetes other than type 2 diabetes, such as type 1 diabetes.

3. Studies that included endodontic treatment for apical periodontitis.

### 2.3. Assessment of Risk of Bias

The risk of bias was evaluated in accordance with the Cochrane Handbook for Systematic Reviews of Interventions, using the following parameters: adequacy of sequence generation; allocation concealment; blinding of participants, personnel, and outcome assessors; incomplete outcome data; and selective outcome reporting [41]. 

### 2.4. Data Extraction

Data containing fundamental information and outcomes, including publication information, country, study design, sample size, participant characteristics, randomization method, allocation concealment, blinding measures, intervention and placebo or control approach, laser parameters and regimen, outcome measurements, follow-up duration, patients lost to follow-up, and the occurrence of any adverse events were collected.

### 2.5. Statistical Analysis

The weight of each study included in the meta-analysis was determined by the reported standard deviation and sample size. The effect size was estimated and reported as the mean difference with a 95% confidence interval (CI) for PPD. Because each analysis included a small number of studies, the variance between the studies was poorly estimated. Thus, a fixed effects model was adopted for the analysis [42]. Heterogeneity was assessed using a chi-square test and I^2^ statistic, at an alpha level of 0.10. The meta-analysis was performed using REVMAN 5.3. For the hypothesis test, an alpha value of 0.05 in a two-tailed Z-test was considered statistically significant.

## 3. Results

### 3.1. Search and Selection Results 

After excluding duplicates from the results of the hand search, 137 reports were identified after the initial search. During the first stage, 124 reports were excluded based on the evaluation of titles and abstracts (inter-reviewer agreement, kappa statistic = 0.92). Second, after screening the full texts of the remaining 13 articles, four reports were excluded for irrelevant outcome measurement [50], insufficient inclusion criteria [40,51], and inappropriate study design [52] (Table S1). Finally, nine eligible reports from eight studies [53,54,55,56,57,58,59,60,61] were included in this systematic review (inter-reviewer agreement, kappa statistic = 0.81). Among the nine reports included, two reports [57,60] had data generated from a single study conducted by Javid et al. [57,60]. Of these, two studies [53,54] with adequate continuous data concerning periodontal health assessed using PPD measurements following melatonin supplementation in combination with non-surgical periodontal therapy, were included in the meta-analysis. 

### 3.2. Characteristics of Included Studies

The study characteristics and parameters related to diabetes and periodontitis are shown in Table 1 and Table 2, respectively. The intervention of antioxidants of included studies includes oral intakes of melatonin [53,54], propolis [55], ascorbic acid (vitamin C) [56,58], resveratrol [57,60], omega-3 fatty acids with cranberry juice [61], and topical use of aloe vera [59] in adjunct to non-surgical periodontal treatment.

### 3.3. Assessment of Methodological Quality

**Figure 1:**
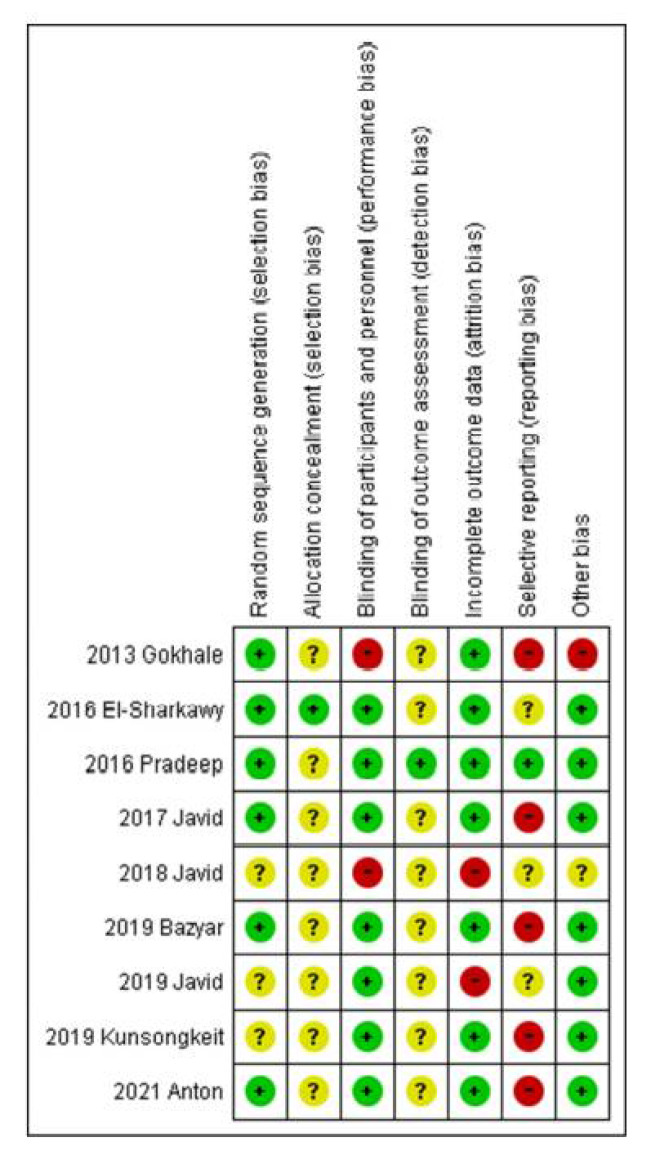
Risk of bias summary: Authors’ judgments about each risk-of-bias item for each included study.

**Figure 2:**
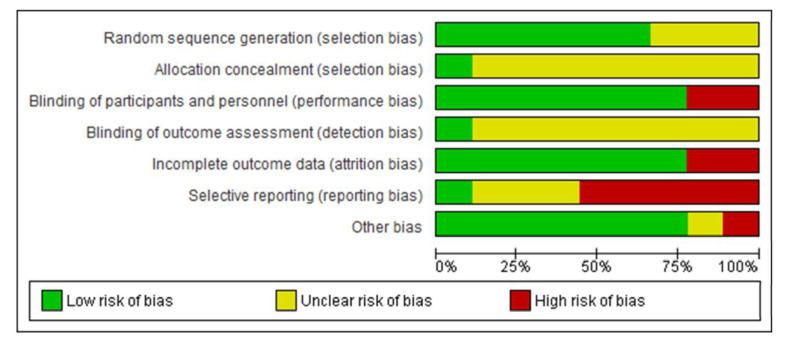
Risk of bias graph: Authors’ judgments about each risk-of-bias item presented as percentages across all included studies.

The results of the methodological quality assessment are shown in Figure 1 and Figure 2. As shown in Figure 1, all studies were assessed as having either a high risk of bias or an unclear risk of bias, although they were presented as RCTs. The randomization used in the included studies were computer-generated random table [55], block design [54,57,60], and coin toss [56,59]. While some studies [53,58,61] did not mention the randomization method. Most studies used a placebo agent and followed a double-blind method for operators, evaluators, and patients [53,54,55,57,58,59,60]. However, the others have an insufficient statement on blinding procedure [56,61]. Among all seven domains, “blinding of outcome assessment” as the detection bias, and “selective reporting” due to the lack of a sufficient statement of the study plan and evaluated parameters, were the principal risk factors affecting the quality of methodology (Figure 2).

### 3.4. Adjunctive Effect of Antioxidants on Periodontal Treatment in Patients with Type 2 Diabetes 

Two melatonin supplementation studies compared SRP alone to SRP plus melatonin therapy (3 mg) over 2 months. Anton et al. showed that the administration of melatonin significantly decreased the mean values of BOP, PPD, and CAL post-intervention [53]. Bazyar et al. showed that melatonin supplementation significantly improved PPD and CAL, but not BOP [54]. Gokhale et al. studied the effects of SRP with ascorbic acid supplementation for 2 weeks and found that dietary ascorbic acid significantly improved the sulcus bleeding index (SBI). However, the improvement in PPD was not statistically significant [56]. Conversely, Kunsongkeit et al. studied the benefit of vitamin C as an adjunct to SRP at 4 and 8 weeks, and showed that SBI, gingival index (GI), PPD, and CAL were significantly different from the baseline. However, no significant difference was found compared to controls at any time interval [58]. Javid et al. reported that resveratrol supplementation along with SRP resulted in a significant improvement in PPD post-intervention compared to SRP alone [60]. Nevertheless, no significant difference in CAL was observed between the intervention and control groups [57]. Another study by the same group evaluated the periodontal status after SRP supplementation with cranberry juice enriched with omega-3 fatty acids in the following four groups: (1) SRP+omega-3, (2) SRP+cranberry juice, (3) SRP+cranberry juice enriched with omega-3, and (4) SRP alone. PPD was significantly reduced in all groups at 8 weeks post-intervention. Moreover, the reduction in PPD was higher in the omega-3 fatty acid group than in the cranberry juice group [61]. El-Sharkawy et al. reported a significantly lower PPD and greater CAL gain in the propolis with SRP group compared to the placebo group at 3 and 6 months after therapy [55]. Furthermore, Pradeep et al. studied the effects of Aloe vera as an adjunct to SRP, and showed that the mean reduction in the plaque index (PI) and modified SBI (mSBI), and the mean gain in CAL, were significantly greater in the Aloe vera group at 3 and 6 months [59].

### 3.5. Meta-Analysis of Adjunctive Application of Melatonin on Periodontal Non-Surgical Therapy

**Figure 3:**
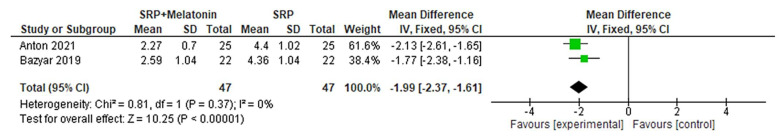
Meta-analysis of adjunctive application of melatonin on periodontal non-surgical therapy; comparison: adjunctive application of melatonin with scaling and root planing versus scaling and root planing alone; outcome: periodontal pocket depth at 8 weeks after the procedure.

A meta-analysis was conducted to assess periodontal disease parameter suppression following the adjunctive application of melatonin in non-surgical periodontal therapy. Two studies [53,54] reported that groups administered with melatonin supplementation (3 mg) showed significantly improved PPD at 8 weeks after SRP compared with the control group. The meta-analysis showed that the adjunctive application group showed significantly improved PPD compared to the control group (mean difference, −1.99; 95% CI, −2.59 to −1.61; *p* < 0.00001) (Figure 3).

## 4. Discussion

In this systematic review, significant improvement in periodontal parameters was reported, compared with SRP alone, following four antioxidant interventions, namely melatonin [53,54], propolis [55], Aloe vera [59], and resveratrol [57,60]. The clinical efficacy of melatonin consumption could be explained by its anti-inflammatory and antioxidant properties, which reduce inflammation in periodontal tissues [62]. Moreover, melatonin has been suggested to have antimicrobial effects against periodontal pathogens [63]. Similarly, the biological effect of propolis probably resulted from a combination of its known antibacterial [64,65], anti-inflammatory, and antioxidant properties [66]. In addition, topical application of propolis as an adjuvant to SRP was more effective than SRP alone, with regard to both microbiological [67] and clinical parameters [45]. The clinical improvement in Aloe vera-supplemented SRP can be attributed to its antibacterial [68] and anti-inflammatory [69] effects on periodontal bacteria, which consequently accelerate collagen synthesis and promote wound healing [70]. Moreover, previous studies have suggested that resveratrol plays a prominent antimicrobial role against periodontal pathogens [71,72] and diminishes oxidative stress by eliminating reactive oxygen species (ROS), enhancing ROS-metabolizing enzymes, and decreasing ROS-producing enzyme activity [73].

In contrast, administration of vitamin C [56,58] and cranberry juice enriched with omega-3 fatty acids [61] resulted in no significant improvement in PPD over SRP alone. Although vitamin C, a well-known antioxidant, can minimize oxidative stress to promote wound healing [74], the findings of clinical trials of vitamin C use as an adjunct to SRP for improving the status of periodontal parameters in diabetes are conflicting [56]. This inconsistency can be explained by the variation in subject characteristics. Patients consuming cranberry juice enriched with omega-3 fatty acids as an adjunct to SRP showed significant improvement post-intervention; however, the effect was not significantly different from that of SRP alone [61]. Relevant findings documented that the inverse relation of consumption of omega-3 fatty acids with periodontitis might be due to their anti-inflammatory effects [75]. One in vitro study using cranberry components demonstrated the inhibition of proinflammatory activity of human gingival fibroblasts exposed to culture conditions similar to physiological conditions of patients with poorly controlled diabetes [76]. A small number of studies have focused on the potential antioxidant and anti-inflammatory effects of cranberry in patients with gingivitis [77]. To date, there have been few studies on the effects of cranberry supplementation, alone or in combination with omega-3 fatty acids, as an adjunct to SRP on periodontal parameters in diabetic patients with periodontitis.

Considering the severity and activity of periodontal disease on antioxidant effects in adjunct with periodontal therapy, deep PPD was reported at the baseline in the studies of vitamin C [56,58] and aloe vera gel [59]. Both studies using vitamin C failed to improve the PPD compared to SRP alone [56,58]. Previous studies suggested that the decreased activity of non-enzymatic antioxidants is linked with periodontitis [78] and that severe periodontal disease is autonomously associated with increased oxidative stress and diminished antioxidant capacity [79]. Conversely, despite the worsening PPD at the baseline compared to other studies, local application of aloe vera in adjunct to SRP could improve periodontal clinical parameters [59]. Thus, whether the severity of periodontal disease might influence the antioxidant activity is still controversial and needs further investigation. Further, although it has been reported that type 2 diabetes was significantly associated with moderate-severe periodontitis among males, but not females, even after adjusting for demographics, socioeconomic status, and oral health behaviors [80]. In this systematic review, none of the studies mentioned the data suggesting the difference between genders nor the response to treatment. Hence, the genders perspective should be one of the interests in a further investigation in diabetic patients with periodontitis.

PPD did not change before and after SRP in the control group of several studies [53,54,60]. As all the subjects included in this study have a type 2 diabetic condition, it might not result from inadequate periodontal treatment that SRP alone could not reach the statistically significant improvement on PPD. Instead, the glycemic status could be a factor to be registered. Thus, a significant reduction in PPD in the intervention groups could explain the mechanisms involved in the beneficial effects of the administered antioxidants in diabetes subjects, with a decrease in inflammatory and oxidative stress markers. 

High HbA1c values are correlated with severe diabetic complications, and reductions in HbA1c levels reduce morbidity and mortality from diabetes [81,82]. All included studies included diabetic individuals with periodontitis, and both diseases have a bidirectional relationship [9,11,12,29]. Of all the included studies, the participants in three studies [53,55,58] had poor glycemic control (HbA1c > 7%) at baseline. However, none mentioned detailed pharmaceutical medications administered during the experiment or any modifications in their medical approach during the experiment. Some randomized clinical trials demonstrated similar results, and were pooled in a meta-analysis, which revealed a mean reduction in HbA1c of approximately 0.40% in patients who received non-surgical periodontal treatment [10,83]. In this systematic review, four articles examined the effects of nutritional interventions on HbA1c levels [53,55,58,61]. Interestingly, three studies reported significant improvement in HbA1c levels with nutritional interventions compared with SRP alone [53,55,61], while the fourth found no significant differences [58]. However, in some of the included studies that did not measure HbA1c levels, other positive changes in systemic parameters such as interleukin (IL)-6 levels [54,57], high-sensitive C-reactive protein levels [54], insulin, and insulin resistance [60] were obtained.

Apart from periodontal disease and type 2 diabetes, other comorbidities, noticeable change in consumption of medications, or any medication that can affect the periodontal status or the beneficial antioxidants intervention were not reported in all the subjects in the included studies. Furthermore, all the studies excluded the use of insulin treatment as an exclusion criterion, except for one study on oral propolis [55] that 8% of all the patients were also using insulin treatment aside from taking oral hypoglycemic drugs. Therefore, insulin therapy could be account for the significant improvements in periodontal parameters and HbA1c outcomes in the study. Similarly, studies in diabetic rodent models revealed that combined insulin and antioxidant therapies resulted in normalization of markers of ROS including plasma MDA, tissue carboxymethyl lysine (CML), and nitrotyrosine [84] and that insulin significantly inhibited neuronal damage through the Nrf2 signaling pathway, which regulates endogenous oxidant-antioxidant balance [85].

Our meta-analysis revealed that groups that underwent melatonin treatment showed significantly improved PPD compared to the control groups [53,54]. Considering the administration period of 8 weeks, it has been suggested that melatonin has antioxidant properties that underlie its potential to reduce inflammation and improve postoperative tissue healing [86].

This systematic review and meta-analysis has several limitations. First, it is desirable to compare antioxidants by type or application protocol, because the mechanisms by which antioxidants act on periodontal tissue might differ depending on the type of antioxidant. However, in this review, because the number of available articles was limited, we did not categorize studies by the type of antioxidant or application protocol. Second, although acceptable, bias assessment indicated that all studies included in this systematic review had methodological weaknesses. Third, the number of available studies was insufficient to confirm the efficacy of each antioxidant as an adjunct to periodontal therapy in patients with type 2 diabetes. The present meta-analysis showed the benefit of adjunctive use of melatonin supplementation in non-surgical periodontal therapy. However, the assessment should be interpreted carefully as only two RCTs could be included in the meta-analysis, and the number of subjects was insufficient. Overall, the small number of included studies contributed to various limitations in this systematic review and meta-analysis. To accumulate evidence regarding the effect of antioxidants in combination with periodontal therapy in patients with type 2 diabetes, more well-designed RCTs with sufficient sample sizes based on the power calculation should be conducted with reference to Cochrane’s risk-of-bias assessment criteria.

## 5. Conclusions

The present systematic review and meta-analysis suggests that the adjunctive use of antioxidants with periodontal therapy significantly suppresses periodontal disease parameters in patients with type 2 diabetes, and indicates that melatonin application combined with non-surgical periodontal therapy might significantly reduce periodontal pocket depth.

## Figures and Tables

**Table 1:** Characteristics of the included studies.

Author (Year)	Country	Age		Participants (Female)		Traceability (%)	Intervention	Evaluation Period	Outcomes in Diabetes	Outcomes in Periodontitis
		Exp	Cont	Exp	Cont					
Anton et al. [53] (2021)	Romania	53.2±3.4	52.2±53	22 (16)	22 (14)	92	1. SRP + melatonin tab (3 mg) 2. SRP + placebo	8 W	HbA1c, waist circumference; hip circumference; waist/hip ratio	PPD, CAL, BOP, PI
Kunsongkeit et al. [58] (2019)	Thailand	59.87 ± 11.3	57.94 ± 14.0	15 (11)	16 (11)	100	1. SRP + Vitamin C (500 mg) 2. SRP + placebo	4, 8 W	FBS, HbA1c	PPD, CAL, PI, SBI
Bazyar et al. [54] (2019)	Iran	53.72 ± 6.68	51.45 ± 5.03	22 (16)	22 (14)	88	1. SRP + melatonin tab (3 mg) 2. SRP + placebo	8 W	height, weight, BMI, waist circumference, hip circumference, waist/hip ratio, physical activity and serum levels of melatonin, IL-6, TNF-α and hc-CRP	PPD, CAL, BOP, PI
Javid et al. [61] (2018)	Iran	1. 57.75 ± 8.58 2. 57.88 ± 6.03 3. 53.14 ± 6.91	53.60 ± 6.23	1. 10 (5) 2. 9 (4) 3. 10 (8)	12 (10)	85	1. SRP + Omega-3 Fatty Acids (180 mg EPA and 120 mg DHA) 2. SRP + Cranberry Juice 3. SRP + Cranberry Juice + Omega-3 Fatty Acids 4. SRP	8 W	Waist circumference, BMI, FBS, HbA1c	PPD
Javid et al. [57,60] (2017, 2019)	Iran	49.1 ± 7.4	50.9±8.9	22 (18)	21 (16)	86	1. SRP + resveratrol (480 mg/day) 2. SRP + placebo capsules	4 W	FBG, Insulin, HOMA-IR, TG, Serum levels of IL6, TNFa and TAC	PPD, CAL
El-Sharkawy et al. [55] (2016)	Egypt	48.9 ± 8.3	51.2 ± 6.5	26 (9)	24 (8)	96	1. SRP + oral propolis (400 mg/day) 2. SRP + placebo	3, 6 M	HbA1c, FPG, serum CML	PPD, CAL, BOP, PI, Eastman interdental bleeding index (EIBI)
Pradeep et al. [59] (2016)	India	35.03 ± 4.78	34.76 ± 5.57	30 (14)	30 (13)	100	1. SRP + locally Aloe vera gel 2. SRP + placebo gel	3, 6 M	N/A	PPD, PI, mSBI, CAL
Gokhale et al. [56] (2013)	India	30–60	30–60	15	15	100	1. SRP + ascorbic acid (450 mg) 2. SRP + placebo candy	2 W	N/A	PPD, PI, SBI

SRP, scaling and root planing; PPD, periodontal pocket depth; CAL, clinical attachment loss; BOP, bleeding on probing; PI, plaque index; GI, gingival index; SBI, sulcus bleeding index; EIBI, Eastman interdental bleeding index; mSBI, modified sulcus bleeding index; HbA1c, Hemoglobin A1C; FBS, fasting blood sugar; BMI, body mass index; HOMA-IR, homeostatic model assessment for insulin resistance; FBG, fasting blood glucose; TG, triglyceride; TAC, total antioxidant capacity; FPG, fasting plasma glucose; CML, Nε-(carboxymethyl) lysine.

**Table 2:** Clinical parameters and antioxidants evaluated in the included studies.

Author (Year)	Intervention	Baseline HbA1c	Baseline PPD	Post HbA1c	Post PPD	Main Findings
Anton et al. [53] (2021)	1. SRP + melatonin tab (3 mg) 2. SRP + placebo	1. 7.6 ± 0.3% 2. 7.6 ± 0.7%	1. 4.65 ± 1.04 mm 2. 4.53 ± 1.01 mm	1. 7.6 ± 0.7% 2. 6.2 ± 0.3%	1. 2.27 ± 0.70 mm 2. 4.40 ± 1.02 mm	Groups that underwent SRP with adjunct melatonin supplementation had significantly improved PPD, CAL, BOP, and HbA1c values when compared to those underwent SRP alone.
Kunsongkeit et al. [58] (2019)	1. SRP + Vitamin C (500 mg) 2. SRP + placebo	1. 7.53 ± 0.79% 2. 8.39 ± 1.5%	1. 5.2 ± 0.41 mm 2. 5.63 ± 1.09 mm	1. 7.27 ± 0.88% 2. 7.98 ± 1.85%	1. (4 W) 3.25 ± 1.09 mm (8 W) 3.25 ± 0.96 mm 2. (4 W) 3.73 ± 1.09 mm (8 W) 3.6 ± 0.9 0mm	Supplementation of vitamin C did not provide an additional benefit with regard to PPD, CAL, PI and SBI.
Bazyar et al. [54] (2019)	1. SRP + melatonin tab (3 mg) 2. SRP + placebo	N/A	1. 4.45 ± 0.96 mm 2. 4.54 ± 1.01 mm	N/A	1. 2.59 ± 1.04 mm 2. 4.36 ± 1.04 mm	Adjunctive melatonin supplementation with SRP significantly improved IL-6 levels, PD, and CAL.
Javid et al. [61] (2018)	1. SRP + Omega-3 Fatty Acids (180 mg EPA and 120 mg DHA) 2. SRP + Cranberry Juice 3. SRP + Cranberry Juice + Omega-3 Fatty Acids 4. SRP	1. 6.82 ± 1.31% 2. 6.17 ± 0.53% 3. 6.32 ± 0.40% 4. 6.64 ± 0.72%	1. 2.5 ± 0.61 mm 2. 2.06 ± 0.54 mm 3. 2.36 ± 0.41 mm 4. 2.42 ± 0.5 mm	1. 5.95 ± 0.60% 2. 5.92 ± 0.65% 3. 5.92 ± 0.19% 4. 6.35 ± 0.76%	1. 1.4 ± 0.28 mm 2. 1.49 ± 0.55 mm 3. 1.59 ± 0.31 mm 4. 1.5 ± 0.45 mm	Cranberry juice enriched with omega-3 fatty acid was beneficial as an adjunctive therapy with SRP in decreasing HbA1c levels, increasing HDL-C levels, and improving PPD.
Javid et al. [57,60] (2017, 2019)	1. SRP + resveratrol (480 mg/day) 2. SRP + placebo capsules	N/A	1. 3.54 ± 0.5 mm 2. 4.06 ± 0.6 mm	N/A	1. 2.35 ± 0.6 mm 2. 3.38 ± 0.5 mm	SRP adjunct to resveratrol supplementation significantly improved PPD, insulin, and HOMA-IR values and serum IL-6 levels, but not TNFa, TAC or CAL levels.
El-Sharkawy et al. [55] (2016)	1. SRP + oral propolis (400 mg/day) 2. SRP + placebo	1. 8.73 ± 0.55% 2. 8.59 ± 0.91%	no data in number	1. (3 M) 7.89 ± 0.43% (6M) 7.75±0.48% 2. (3 M) 8.58 ± 0.82% (6M) 8.5 ± 0.73%	no data in number	Groups that underwent SRP adjunct to propolis supplementation showed significantly improved PPD, CAL and HbA1c values compared to the groups of SRP alone.
Pradeep et al. [59] (2016)	1. SRP + locally Aloe vera gel 2. SRP + placebo gel	N/A	1. 7.26 ± 0.94 mm 2. 7.27 ± 0.92 mm	N/A	1. (3 M) 5.43 ± 0.97 mm (6 M) 4.60 ± 0.93 mm 2. (3 M) 6.10 ± 0.99 mm (6 M) 5.27 ± 0.98 mm	SRP adjunct to aloe vera gel induced significantly greater reductions in PI, mSBI, PD and gain in CAL compared to SRP adjunct to placebo gel.
Gokhale et al. [56] (2013)	1. SRP + ascorbic acid (450 mg) 2. SRP + placebo candy	N/A	1. 6.93 ± 1.63 mm 2. 7.11 ± 1.50 mm	N/A	1. 6.26 ± 1.34 mm 2. 6.61 ± 1.62 mm	SRP adjunct to ascorbic acid supplementation significantly improved SBI. However, improvement in PPD was not statistically significant.

SRP, scaling and root planing; PPD, periodontal pocket depth; CAL, clinical attachment loss; BOP, bleeding on probing; PI, plaque index; SBI, sulcus bleeding index; mSBI, modified sulcus bleeding index; HbA1c, hemoglobin A1C; HOMA-IR, homeostatic model assessment for insulin resistance; TAC, total antioxidant capacity; HDL-C, high-density lipoprotein cholesterol.

## Supplementary Materials

The following are available online at https://www.mdpi.com/article/10.3390/antiox10081304/s1, Table S1: Excluded investigations.
