# Supplementary material for: Effects of Antioxidant in Adjunct with Periodontal Therapy in Patients with Type 2 Diabetes: A Systematic Review and Meta-Analysis
Source: Antioxidants (Basel). 2021 Aug 18;10(8):1304. doi: 10.3390/antiox10081304 (PMC8389262; doi:10.3390/antiox10081304)
Supplement: Supplementary file 1 [file antioxidants-10-01304-s001.zip › antioxidants-1266141-supplementary.pdf]

Table S1. Excluded investigations

Author (Year)	Reason
Javid et al. [50] (2020)	Irrelevant outcome measurement; no periodontal parameters measurement. Other analysis of the previous study[32].
Montero et al. [51] (2017)	Insufficient inclusion criteria; not performed periodontal non-surgical therapy such as scaling and root planing
Cutando et al. [40] (2015)	Insufficient inclusion criteria; comparing between diabetes and healthy patients. Other analysis of the previous study[28].
Amaliya et al. [52] (2015)	Prospective cohort study, not RCT
